# Editorial: Emerging Arboviruses

**DOI:** 10.3389/fvets.2020.593872

**Published:** 2020-11-06

**Authors:** William C. Wilson, Dana Mitzel, Giovanni Savini, Stéphan Zientara, Juergen A. Richt

**Affiliations:** ^1^Arthropod-Borne Animal Diseases Research Unit, United States Department of Agriculture (USDA), Agricultural Research Service, Manhattan, KS, United States; ^2^OIE Reference Laboratory for Bluetongue, IZS Istituto Zooprofilattico Sperimentale, Teramo, Italy; ^3^French Agency for Food, Environmental and Occupational Health & Safety (ANSES), Maisons Alfort, France; ^4^Department of Diagnostic Medicine/Pathobiology and Center of Excellence for Emerging and Zoonotic Animal Diseases, College of Veterinary Medicine, Kansas State University, Manhattan, KS, United States

**Keywords:** emerging, arbovirus, diagnostic, vaccine, risk model

## Introduction

The current COVID-19 pandemic has highlighted the well-known fact that debilitating infectious diseases can emerge naturally from an animal source. The estimates range from 60 to 75% of emerging infectious diseases in humans are of zoonotic origin. Up to one third of these emerging infectious diseases are caused by vector-borne pathogens, which cause more than 700,000 deaths annually (1). In the last two decades, there are multiple examples of the emergence of vector-borne pathogens affecting both animal and public health. This includes animal pathogens such as the introduction and re-emergence of Bluetongue virus serotype 8 into ruminant livestock in Europe (2) and the introduction of African Swine fever virus to Europe and Asia (Gaudreault et al.). On the zoonotic side, there is West Nile virus that was introduced into the United States in 1999 (3), Rift Valley fever which emerged in the Arabian Peninsula outside of its endemic area in Sub-Saharan Africa (4), and the displacement of one genotype of Japanese encephalitis virus by another in Asia (5) as well as its potential emergence in Australia (6); these are all examples of the recent emergence of arboviruses into animal and human populations. Emerging or re-emerging vector-borne diseases are an important *Global One Health* concern. A detailed understanding of the virus-vector-host interactions in its natural environment is critical to develop effective diagnostics, vaccines, and other control strategies. The present collection of manuscripts was developed to provide an unique compilation of recent advances in research and mitigation strategies for emerging and re-emerging arboviruses of veterinary/agricultural and public health concern such as viruses from the families *Asfarviriadae, Flaviviridae, Phenuiviridae, Reoviridae*, and *Togaviridae*. This manuscript collection will provide scientists up-to-date information on these diverse pathogens and their respective insect vectors. It is organized with an initial series of articles reviewing risk assessments, which is followed by articles reviewing the state-of-the-art in epidemiology, diagnostics, vaccines, and other control methods.

## Organization of the Special Edition

This Special Edition includes three several comprehensive reviews describing various emerging arthropod-borne disease threats in animals, each from a different continent with a different perspective. The first review is provided by Folly et al. and provides an overview of mosquitoes and mosquito-borne diseases and their possible risk of introduction into the UK. It also discusses ticks and tick-borne diseases including Louping ill virus, which is endemic in the UK, and biting midge-borne viruses, two of which emerged in Europe since 2006. The need for surveillance of these pathogens in domestic pets, livestock and wildlife is emphasized. The second review by Yanase et al. addresses endemic and emerging mosquito, tick-, and midge-borne arboviruses that affect domestic ruminants in East Asia. The midge-borne arboviruses include Akabane, Aino, and Chuzan viruses associated with reproductive losses in domestic ruminants in East Asia. The relevance in other Culicoides-transmitted viruses such as Bluetongue and the emergence of Epizootic Hemorrhagic Disease virus, serotype 6, in East Asia and Japan are also discussed. The final emerging disease review by Kading et al. identifies potential pathogen threats of concern to the United States. African Swine fever, Japanese encephalitis and Rift Valley fever as the top three arboviral threats are discussed in detail. In addition, currently available surveillance and diagnostic tools are summarized including useful information for the development of detection and response plans for these arboviral threats.

The above described reviews are followed by a series of manuscripts which are described below in virus-based alphabetic order. The review on the emerging DNA arbovirus, African Swine fever virus (ASFV), by Gaudreault et al. summarizes the threat of this pathogen to the swine industry worldwide. ASF has been a concern in Africa for more than a century and first emerged in Europe in the 1950s and again in the 1960s, and to Russia, Caribbean Islands and South America in the 1970's; it was controlled by the mid-1990's. The second emergence started in 2007 in the Caucasus region and since then the virus has spread across Eastern Europe and Southeast Asia. This review provides an overview of the epidemiology, biology, diagnostic, and mitigation strategies for this rather complex arboviral threat pathogen.

The next series of manuscripts describe various aspects of Bluetongue virus (BTV) that a long history of causing disease and economic losses. A description of the clinical disease associated with the incursion of BTV serotype 3 in Israel is provided by Golender et al., and the detection and characterization of BTV serotype 14 in Russia by Koltsov et al.. In 2006, BTV serotype 8 was introduced into Europe and caused substantial clinical disease and also transplacental virus transmission. The failure to remove BTV serotype 8 from bovine embryos with subsequent spread of the virus via embryo transfers demonstrated the need for screening of embryo donors and embryos for BTV in endemic areas (Haegeman et al.). This is critical since embryo transfer techniques have been used as a way to safely transfer genetic materials from a BTV endemic area to non-endemic regions. Since 1999, 11 exotic BTV serotypes have been isolated in the Southeastern United States but only one has been found outside of the Southeastern region (7). An overview of the ecology and epidemiology of BTV in North America by Mayo et al. provides insights into the environmental factors that drive virus transmission. Sensitive and early detection of BTV is essential to rapidly detect BTV and to facilitate mitigation strategies. Rocchigiani et al. describe the development of sensitive digital RT-qPCR for accurate quantification of BTV in field samples. This field-deployable RT-qPCR assay demonstrates similar sensitivity and specificity to a previously established laboratory RT-qPCR assay.

Flaviviruses are discussed in the next series of manuscripts including Japanese encephalitis, Tembusu, Usutu, West Nile, and Zika viruses. Endemic circulation of Japanese encephalitis virus (JEV) in Southeast Asia is well-established; this is also an area where West Nile virus (WNV) is known to circulate. A seroepidemiolgical study by Auerswald et al. demonstrates clear evidence of current circulation of these viruses in Cambodian domestic birds; these findings necessitate the need for increased surveillance for these viruses in this region. The factors affecting the risks of JEV introduction into the United States were assessed by Oliveira et al.; this was done by qualitative risk assessment. WNV emerged in Europe in the early 2000's and another flavivirus, Usutu virus (USUV), which was originally found in Africa, was identified for the first time in the 1996 in Italy. Vilibic-Cavlek et al. summarize the epidemiology of these two mosquito-borne flaviviruses (WNV and USUV) in Southern Europe. Entomological surveillance by Calzolari et al. demonstrates increased circulation of WNV in Northern Italy and the importance of temperature on WNV infection of mosquitoes. A less known flavivirus, Tembusu virus which causes an egg-drop syndrome in ducks, emerged in 2010 in China. Vaccines against Tembusu virus have been generated but understanding of the neutralizing immune response to infection or vaccination is minimal. Lv et al. describe the development of a plaque reduction neutralization titration assay to detect antibodies to Tembusu virus and found long-lasting neutralizing antibodies in sera from infected and vaccinated flocks. The Japanese serocomplex includes JEV, USUV and WNV; whereas, Tembusu is grouped within the Ntaya serocomplex. The Ntaya complex also includes the Bagaza/Israel turkey meningoencephalomyelitis virus. The development of a duplex RT-qPCR assay to detect and distinguish between the Japanese and Ntaya serocomplexes is reported by Elizalde et al.. Zika virus is the most recent emerging mosquito-borne virus for which there are limited animal models available. Ambagala et al. conducted experimental infections with Zika virus in cattle, chickens, pigs, sheep and chicken embryos. None of the animals were susceptible to experimental infection except for the chicken embryos; this could provide an additional tool for Zika virus investigations.

The next series of manuscripts focusses on Rift Valley fever (RVF) which is a mosquito-borne zoonotic disease endemic in Sub-Saharan Africa. RVF is a significant public and animal health concern and reliable RVF virus (RVFV) infection models are badly needed to develop advanced diagnostic tools and control strategies. A review on presently available livestock models (cattle, sheep and goats) for experimental RVF virus infections is provided by Kroeker et al. Although RVFV is primarily transmitted by mosquito bites it is known that it can also be transmitted by aerosol (8). In addition, contact transmission has been demonstrated in one study of experimental RVFV infection of white-tailed deer (9). A comparison of different routes of experimental RVFV infection is reported by Kroeker et al.. Approaches to RVFV diagnostics and vaccines have been reviewed previously (10, 11); however, a perhaps less known vector-based vaccine approach for RVFV is using capripoxvirus; the use of this virus vector for vaccines against different arboviruses affecting ruminant livestock is addressed in a review by Teffera and Babiuk. A similar approach is described by Wallace et al. with the development of a bivalent Lumpy Skin Disease-vectored Rift Valley fever virus vaccine.

An important part of a successful mitigation strategy for emerging and endemic pathogens is the ability to detect the presence of the agent in a rapid, sensitive and specific manner. The final paper in this Special Edition describes a novel approach using nanoparticles to preserve arboviral RNA in blood samples of infected animals thus increasing the ability for detection. Akhrymuk et al. discuss the development of this approach using Venezuelan equine encephalitis virus (VEEV), an often neglected but important zoonotic viral pathogen that can cause high mortality in horses.

## Conclusion

The emergence of a new viral disease could affect public and animal health as well as the economy of many countries worldwide as demonstrated by the current COVID-19 pandemic. Therefore, we cannot forget that other known and unknown animal and zoonotic pathogens could also pose considerable threats to animal and public health globally. Arthropod-borne pathogens have emerged and are re-emerging in increased numbers in the last decades. This Special Edition provides overviews of these arboviral threats and discusses current and novel strategies for their detection and control.

## Author Contributions

WW, DM, and JR wrote the editorial with careful editing and comments from SZ and GS. All authors contributed to the article and approved the submitted version.

## Conflict of Interest

The authors declare that the research was conducted in the absence of any commercial or financial relationships that could be construed as a potential conflict of interest.
